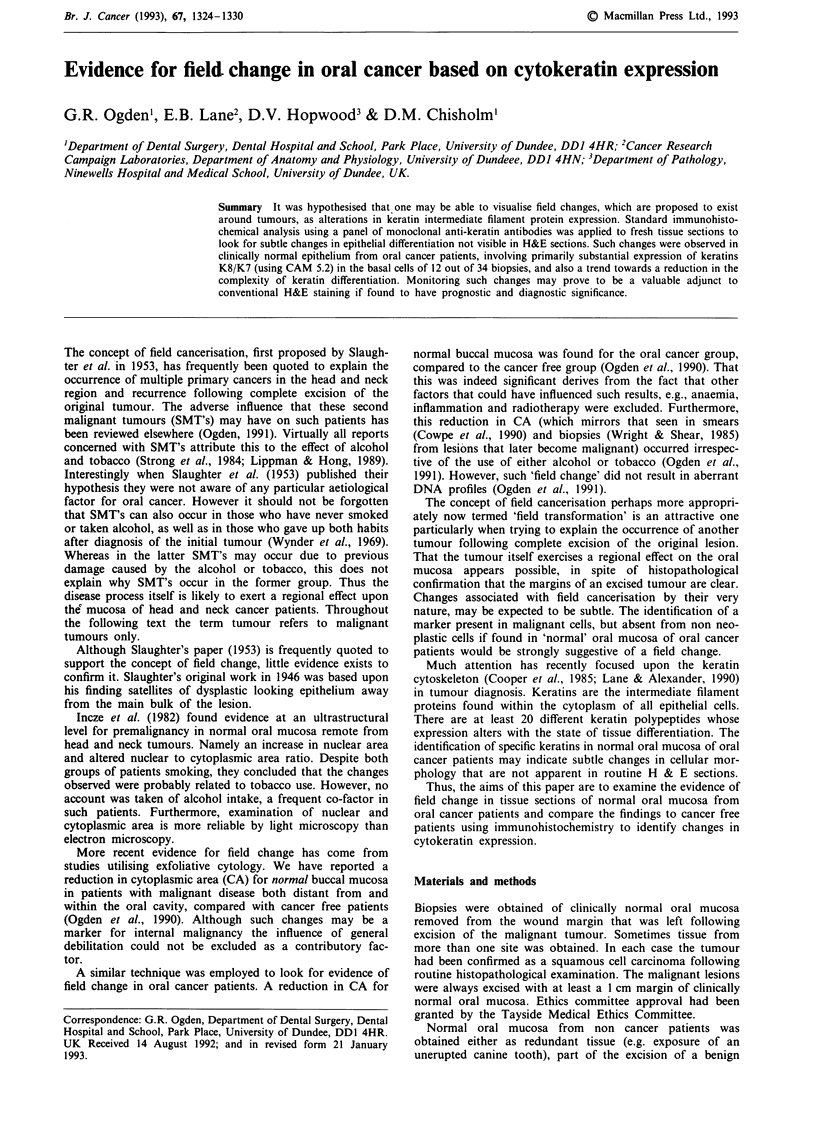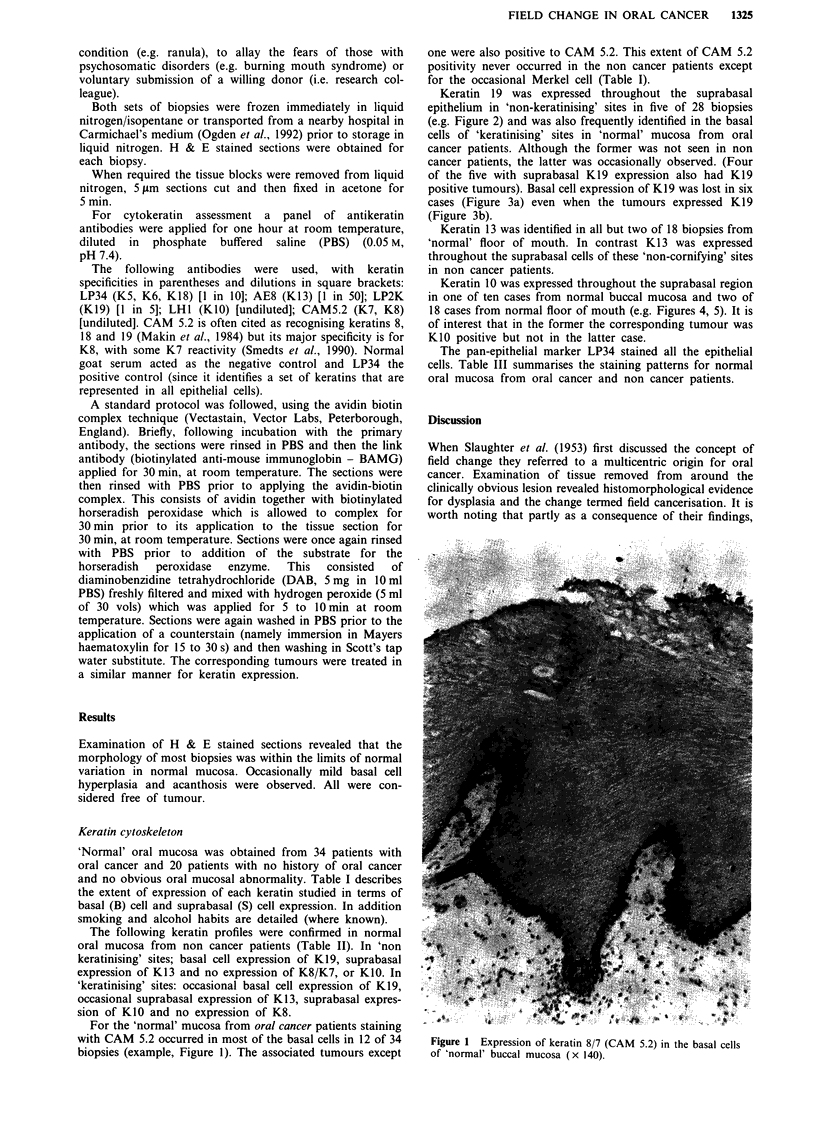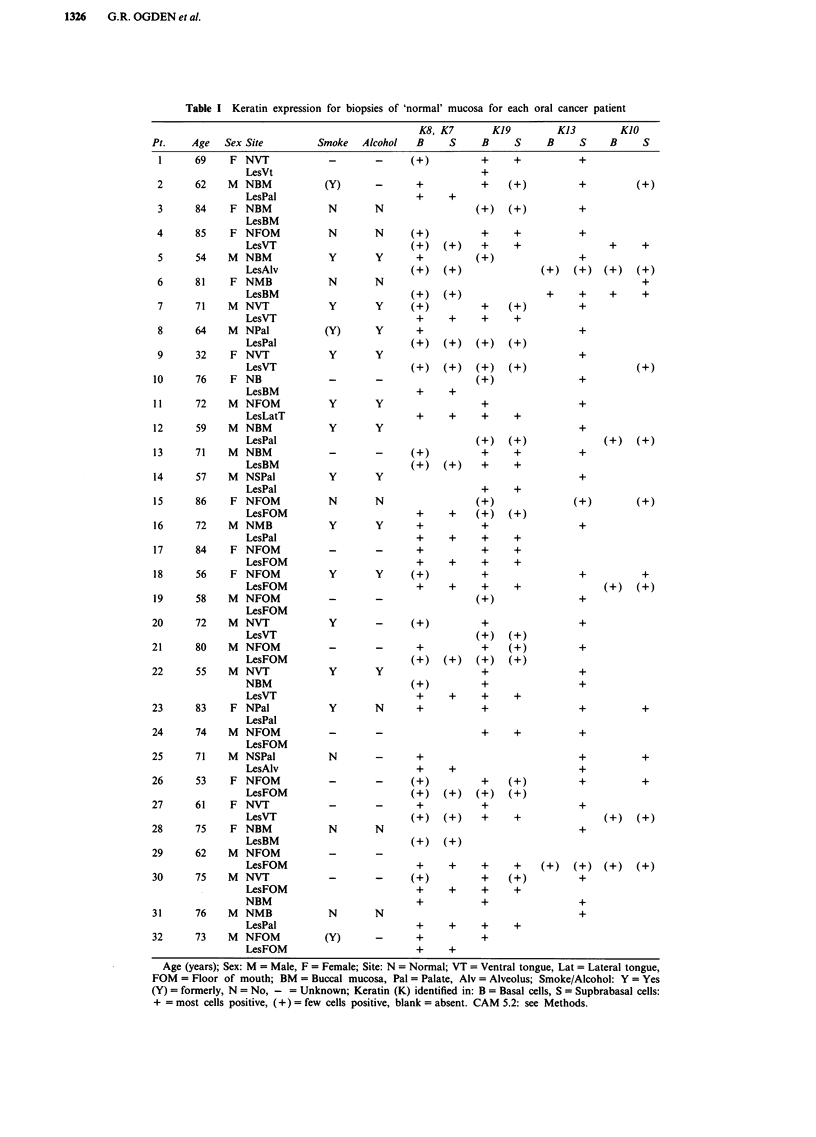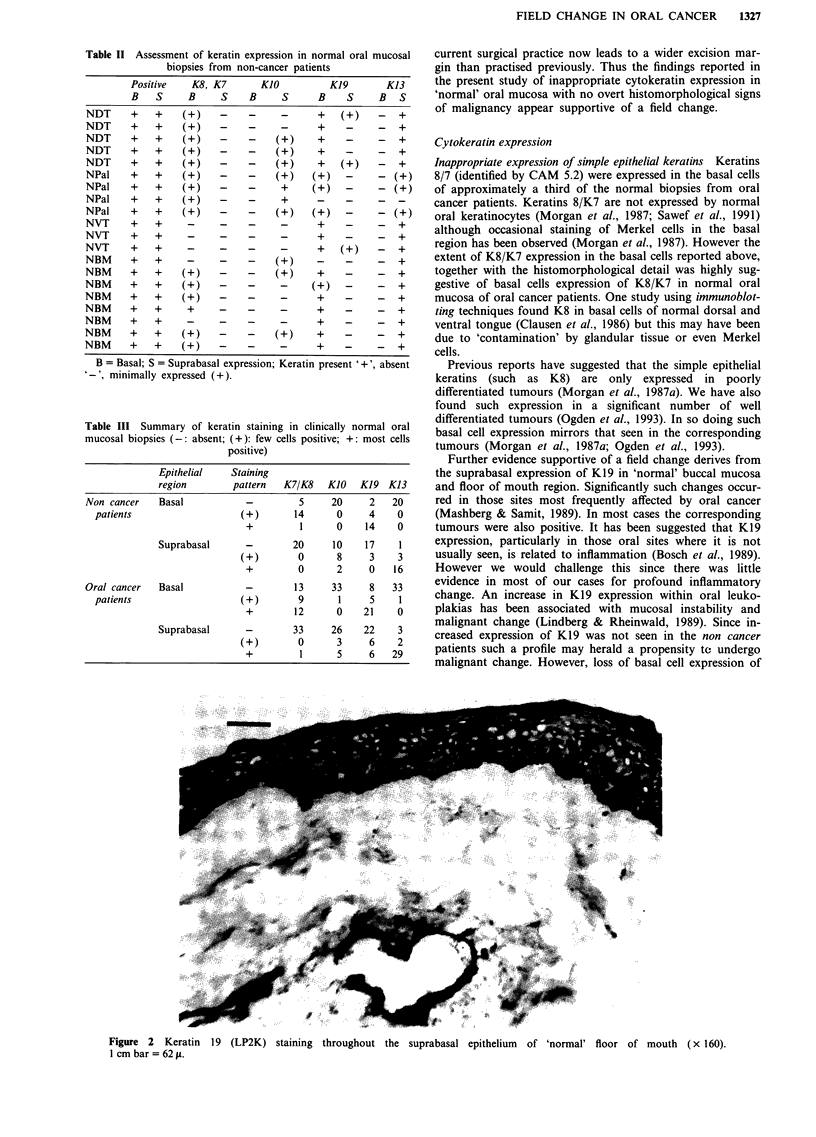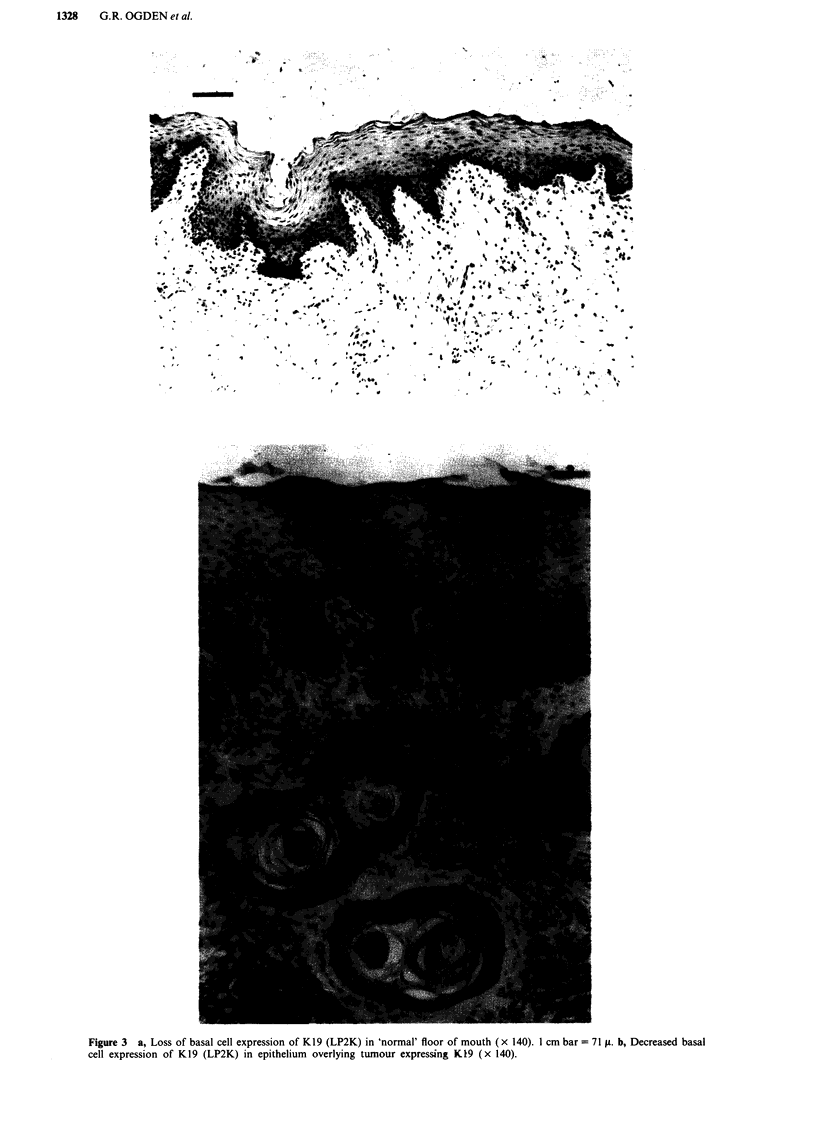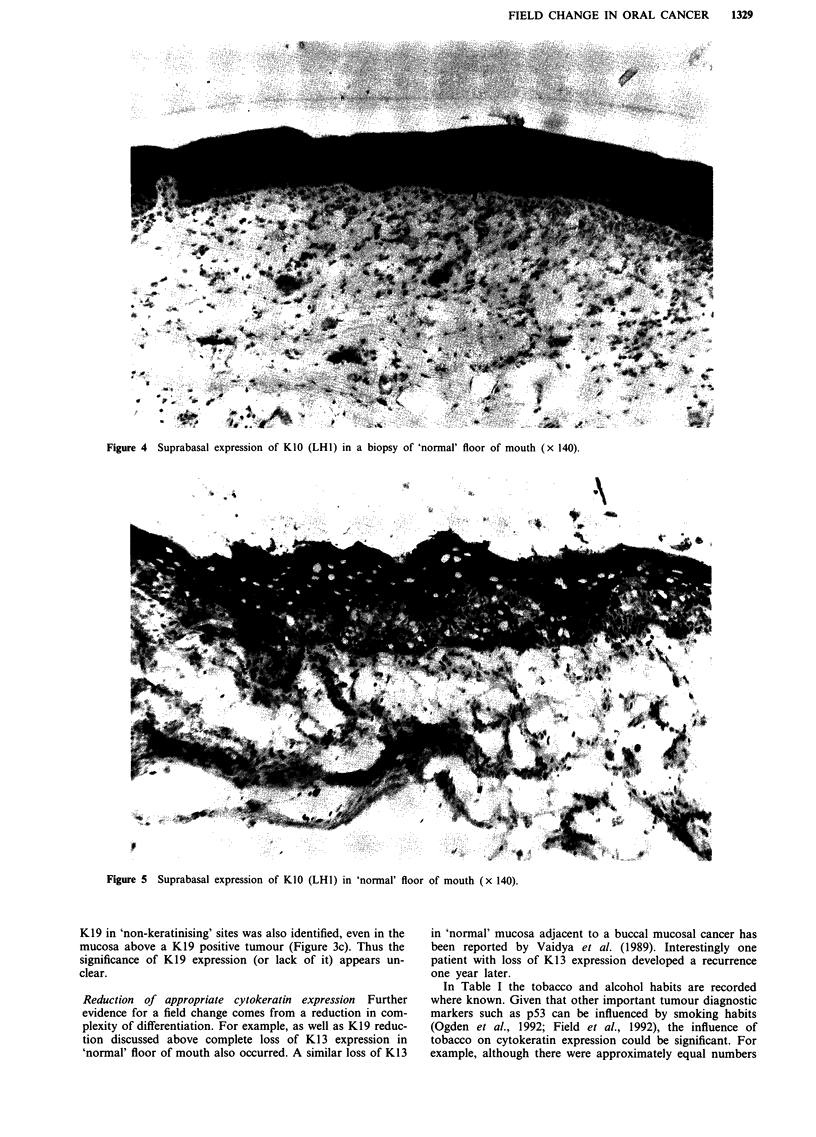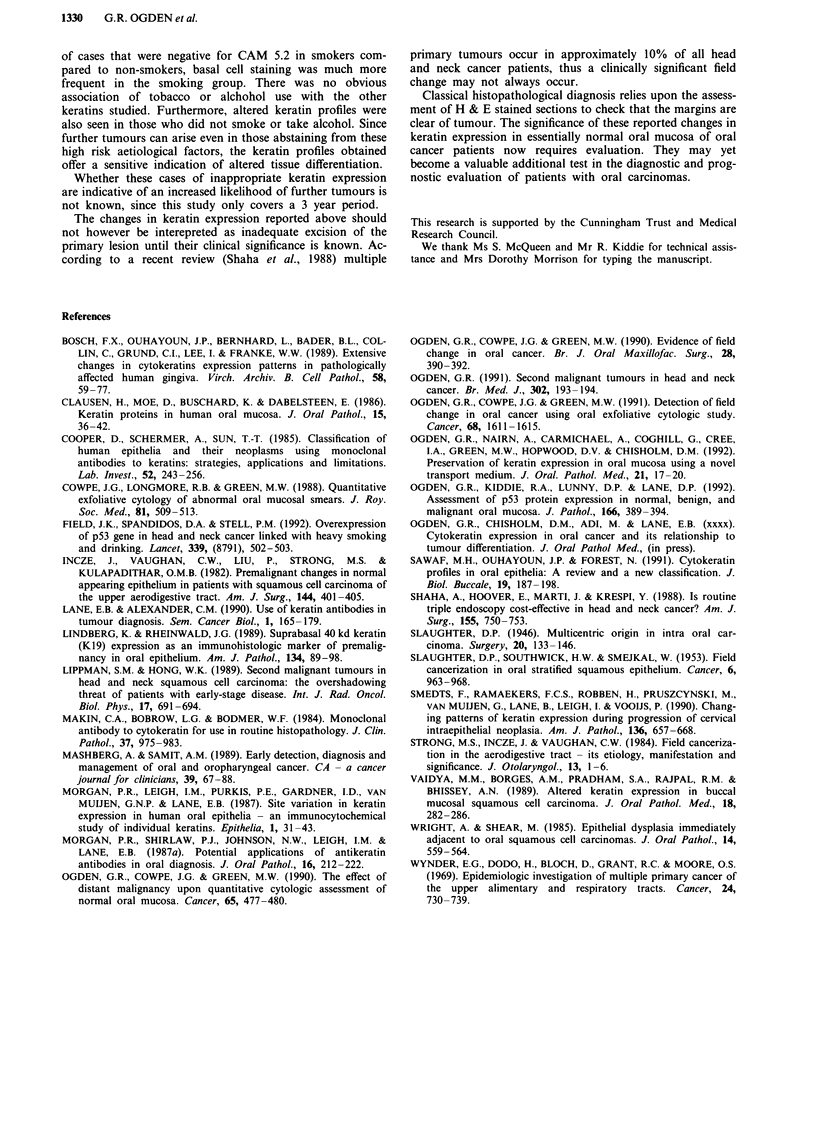# Evidence for field change in oral cancer based on cytokeratin expression.

**DOI:** 10.1038/bjc.1993.245

**Published:** 1993-06

**Authors:** G. R. Ogden, E. B. Lane, D. V. Hopwood, D. M. Chisholm

**Affiliations:** Department of Dental Surgery, Dental Hospital and School, University of Dundee, UK.

## Abstract

**Images:**


Br. J. Cancer (1993), 67, 1324–1330 © Macmillan Press Ltd., 1993

### Evidence for field change in oral cancer based on cytokeratin expression

G.R. Ogden[1], E.B. Lane[2], D.V. Hopwood[3] & D.M. Chisholm[1]

[1]*Department of Dental Surgery, Dental Hospital and School, Park Place, University of Dundee, DD1 4HR;* [2]*Cancer Research Campaign Laboratories, Department of Anatomy and Physiology, University of Dundeee, DD1 4HN;* [3]*Department of Pathology, Ninewells Hospital and Medical School, University of Dundee, UK.*

**Summary** It was hypothesised that one may be able to visualise field changes, which are proposed to exist around tumours, as alterations in keratin intermediate filament protein expression. Standard immunohistochemical analysis using a panel of monoclonal anti-keratin antibodies was applied to fresh tissue sections to look for subtle changes in epithelial differentiation not visible in H&E sections. Such changes were observed in clinically normal epithelium from oral cancer patients, involving primarily substantial expression of keratins K8/K7 (using CAM 5.2) in the basal cells of 12 out of 34 biopsies, and also a trend towards a reduction in the complexity of keratin differentiation. Monitoring such changes may prove to be a valuable adjunct to conventional H&E staining if found to have prognostic and diagnostic significance.

The concept of field cancerisation, first proposed by Slaughter *et al.* in 1953, has frequently been quoted to explain the occurrence of multiple primary cancers in the head and neck region and recurrence following complete excision of the original tumour. The adverse influence that these second malignant tumours (SMT's) may have on such patients has been reviewed elsewhere (Ogden, 1991). Virtually all reports concerned with SMT's attribute this to the effect of alcohol and tobacco (Strong *et al.*, 1984; Lippman & Hong, 1989). Interestingly when Slaughter *et al.* (1953) published their hypothesis they were not aware of any particular aetiological factor for oral cancer. However it should not be forgotten that SMT's can also occur in those who have never smoked or taken alcohol, as well as in those who gave up both habits after diagnosis of the initial tumour (Wynder *et al.*, 1969). Whereas in the latter SMT's may occur due to previous damage caused by the alcohol or tobacco, this does not explain why SMT's occur in the former group. Thus the disease process itself is likely to exert a regional effect upon the mucosa of head and neck cancer patients. Throughout the following text the term tumour refers to malignant tumours only.

Although Slaughter's paper (1953) is frequently quoted to support the concept of field change, little evidence exists to confirm it. Slaughter's original work in 1946 was based upon his finding satellites of dysplastic looking epithelium away from the main bulk of the lesion.

Incze *et al.* (1982) found evidence at an ultrastructural level for premalignancy in normal oral mucosa remote from head and neck tumours. Namely an increase in nuclear area and altered nuclear to cytoplasmic area ratio. Despite both groups of patients smoking, they concluded that the changes observed were probably related to tobacco use. However, no account was taken of alcohol intake, a frequent co-factor in such patients. Furthermore, examination of nuclear and cytoplasmic area is more reliable by light microscopy than electron microscopy.

More recent evidence for field change has come from studies utilising exfoliative cytology. We have reported a reduction in cytoplasmic area (CA) for *normal* buccal mucosa in patients with malignant disease both distant from and within the oral cavity, compared with cancer free patients (Ogden *et al.*, 1990). Although such changes may be a marker for internal malignancy the influence of general debilitation could not be excluded as a contributory factor.

A similar technique was employed to look for evidence of field change in oral cancer patients. A reduction in CA for normal buccal mucosa was found for the oral cancer group, compared to the cancer free group (Ogden *et al.*, 1990). That this was indeed significant derives from the fact that other factors that could have influenced such results, e.g., anaemia, inflammation and radiotherapy were excluded. Furthermore, this reduction in CA (which mirrors that seen in smears (Cowpe *et al.*, 1990) and biopsies (Wright & Shear, 1985) from lesions that later become malignant) occurred irrespective of the use of either alcohol or tobacco (Ogden *et al.*, 1991). However, such 'field change' did not result in aberrant DNA profiles (Ogden *et al.*, 1991).

The concept of field cancerisation perhaps more appropriately now termed 'field transformation' is an attractive one particularly when trying to explain the occurrence of another tumour following complete excision of the original lesion. That the tumour itself exercises a regional effect on the oral mucosa appears possible, in spite of histopathological confirmation that the margins of an excised tumour are clear. Changes associated with field cancerisation by their very nature, may be expected to be subtle. The identification of a marker present in malignant cells, but absent from non neoplastic cells if found in 'normal' oral mucosa of oral cancer patients would be strongly suggestive of a field change.

Much attention has recently focused upon the keratin cytoskeleton (Cooper *et al.*, 1985; Lane & Alexander, 1990) in tumour diagnosis. Keratins are the intermediate filament proteins found within the cytoplasm of all epithelial cells. There are at least 20 different keratin polypeptides whose expression alters with the state of tissue differentiation. The identification of specific keratins in normal oral mucosa of oral cancer patients may indicate subtle changes in cellular morphology that are not apparent in routine H & E sections.

Thus, the aims of this paper are to examine the evidence of field change in tissue sections of normal oral mucosa from oral cancer patients and compare the findings to cancer free patients using immunohistochemistry to identify changes in cytokeratin expression.

### Materials and methods

Biopsies were obtained of clinically normal oral mucosa removed from the wound margin that was left following excision of the malignant tumour. Sometimes tissue from more than one site was obtained. In each case the tumour had been confirmed as a squamous cell carcinoma following routine histopathological examination. The malignant lesions were always excised with at least a 1 cm margin of clinically normal oral mucosa. Ethics committee approval had been granted by the Tayside Medical Ethics Committee.

Normal oral mucosa from non cancer patients was obtained either as redundant tissue (e.g. exposure of an unerupted canine tooth), part of the excision of a benign

Correspondence: G.R. Ogden, Department of Dental Surgery, Dental Hospital and School, Park Place, University of Dundee, DD1 4HR. UK Received 14 August 1992; and in revised form 21 January 1993.

condition (e.g. ranula), to allay the fears of those with psychosomatic disorders (e.g. burning mouth syndrome) or voluntary submission of a willing donor (i.e. research colleague).

Both sets of biopsies were frozen immediately in liquid nitrogen/isopentane or transported from a nearby hospital in Carmichael's medium (Ogden *et al.*, 1992) prior to storage in liquid nitrogen. H & E stained sections were obtained for each biopsy.

When required the tissue blocks were removed from liquid nitrogen, 5 μm sections cut and then fixed in acetone for 5 min.

For cytokeratin assessment a panel of antikeratin antibodies were applied for one hour at room temperature, diluted in phosphate buffered saline (PBS) (0.05 M, pH 7.4).

The following antibodies were used, with keratin specificities in parentheses and dilutions in square brackets: LP34 (K5, K6, K18) [1 in 10]; AE8 (K13) [1 in 50]; LP2K (K19) [1 in 5]; LH1 (K10) [undiluted]; CAM5.2 (K7, K8) [undiluted]. CAM 5.2 is often cited as recognising keratins 8, 18 and 19 (Makin *et al.*, 1984) but its major specificity is for K8, with some K7 reactivity (Smedts *et al.*, 1990). Normal goat serum acted as the negative control and LP34 the positive control (since it identifies a set of keratins that are represented in all epithelial cells).

A standard protocol was followed, using the avidin biotin complex technique (Vectastain, Vector Labs, Peterborough, England). Briefly, following incubation with the primary antibody, the sections were rinsed in PBS and then the link antibody (biotinylated anti-mouse immunoglobin – BAMG) applied for 30 min, at room temperature. The sections were then rinsed with PBS prior to applying the avidin-biotin complex. This consists of avidin together with biotinylated horseradish peroxidase which is allowed to complex for 30 min prior to its application to the tissue section for 30 min, at room temperature. Sections were once again rinsed with PBS prior to addition of the substrate for the horseradish peroxidase enzyme. This consisted of diaminobenzidine tetrahydrochloride (DAB, 5 mg in 10 ml PBS) freshly filtered and mixed with hydrogen peroxide (5 ml of 30 vols) which was applied for 5 to 10 min at room temperature. Sections were again washed in PBS prior to the application of a counterstain (namely immersion in Mayers haematoxylin for 15 to 30 s) and then washing in Scott's tap water substitute. The corresponding tumours were treated in a similar manner for keratin expression.

### Results

Examination of H & E stained sections revealed that the morphology of most biopsies was within the limits of normal variation in normal mucosa. Occasionally mild basal cell hyperplasia and acanthosis were observed. All were considered free of tumour.

### *Keratin cytoskeleton*

'Normal' oral mucosa was obtained from 34 patients with oral cancer and 20 patients with no history of oral cancer and no obvious oral mucosal abnormality. Table I describes the extent of expression of each keratin studied in terms of basal (B) cell and suprabasal (S) cell expression. In addition smoking and alcohol habits are detailed (where known).

The following keratin profiles were confirmed in normal oral mucosa from non cancer patients (Table II). In 'non keratinising' sites; basal cell expression of K19, suprabasal expression of K13 and no expression of K8/K7, or K10. In 'keratinising' sites: occasional basal cell expression of K19, occasional suprabasal expression of K13, suprabasal expression of K10 and no expression of K8.

For the 'normal' mucosa from *oral cancer* patients staining with CAM 5.2 occurred in most of the basal cells in 12 of 34 biopsies (example, Figure 1). The associated tumours except one were also positive to CAM 5.2. This extent of CAM 5.2 positivity never occurred in the non cancer patients except for the occasional Merkel cell (Table I).

Keratin 19 was expressed throughout the suprabasal epithelium in 'non-keratinising' sites in five of 28 biopsies (e.g. Figure 2) and was also frequently identified in the basal cells of 'keratinising' sites in 'normal' mucosa from oral cancer patients. Although the former was not seen in non cancer patients, the latter was occasionally observed. (Four of the five with suprabasal K19 expression also had K19 positive tumours). Basal cell expression of K19 was lost in six cases (Figure 3a) even when the tumours expressed K19 (Figure 3b).

Keratin 13 was identified in all but two of 18 biopsies from 'normal' floor of mouth. In contrast K13 was expressed throughout the suprabasal cells of these 'non-cornifying' sites in non cancer patients.

Keratin 10 was expressed throughout the suprabasal region in one of ten cases from normal buccal mucosa and two of 18 cases from normal floor of mouth (e.g. Figures 4, 5). It is of interest that in the former the corresponding tumour was K10 positive but not in the latter case.

The pan-epithelial marker LP34 stained all the epithelial cells. Table III summarises the staining patterns for normal oral mucosa from oral cancer and non cancer patients.

### Discussion

When Slaughter *et al.* (1953) first discussed the concept of field change they referred to a multicentric origin for oral cancer. Examination of tissue removed from around the clinically obvious lesion revealed histomorphological evidence for dysplasia and the change termed field cancerisation. It is worth noting that partly as a consequence of their findings,

**Figure 1** Expression of keratin 8/7 (CAM 5.2) in the basal cells of 'normal' buccal mucosa (× 140).

**Table I** Keratin expression for biopsies of 'normal' mucosa for each oral cancer patient

| | | | | | | *K8, K7* | | *K19* | | *K13* | | *K10* | |
|---|---|---|---|---|---|---|---|---|---|---|---|---|---|
| *Pt.* | *Age* | *Sex* | *Site* | *Smoke* | *Alcohol* | *B* | *S* | *B* | *S* | *B* | *S* | *B* | *S* |
| 1 | 69 | F | NVT | – | – | (+) | | + | + | | + | | |
| | | | LesVt | | | | | + | | | | | |
| 2 | 62 | M | NBM | (Y) | – | + | | + | (+) | | + | | (+) |
| | | | LesPal | | | + | + | | | | | | |
| 3 | 84 | F | NBM | N | N | | | (+) | (+) | | + | | |
| | | | LesBM | | | | | | | | | | |
| 4 | 85 | F | NFOM | N | N | (+) | | + | + | | + | | |
| | | | LesVT | | | (+) | (+) | + | + | | | + | + |
| 5 | 54 | M | NBM | Y | Y | + | | (+) | | | + | | |
| | | | LesAlv | | | (+) | (+) | | | (+) | (+) | (+) | (+) |
| 6 | 81 | F | NMB | N | N | | | | | | | | + |
| | | | LesBM | | | (+) | (+) | | | + | + | + | + |
| 7 | 71 | M | NVT | Y | Y | (+) | | + | (+) | | + | | |
| | | | LesVT | | | + | + | + | + | | | | |
| 8 | 64 | M | NPal | (Y) | Y | + | | | | | + | | |
| | | | LesPal | | | (+) | (+) | (+) | (+) | | | | |
| 9 | 32 | F | NVT | Y | Y | | | | | | + | | |
| | | | LesVT | | | (+) | (+) | (+) | (+) | | | | (+) |
| 10 | 76 | F | NB | – | – | | | (+) | | | + | | |
| | | | LesBM | | | + | + | | | | | | |
| 11 | 72 | M | NFOM | Y | Y | | | + | | | + | | |
| | | | LesLatT | | | + | + | + | + | | | | |
| 12 | 59 | M | NBM | Y | Y | | | | | | + | | |
| | | | LesPal | | | | | (+) | (+) | | | (+) | (+) |
| 13 | 71 | M | NBM | – | – | (+) | | + | + | | + | | |
| | | | LesBM | | | (+) | (+) | + | + | | | | |
| 14 | 57 | M | NSPal | Y | Y | | | | | | + | | |
| | | | LesPal | | | | | + | + | | | | |
| 15 | 86 | F | NFOM | N | N | | | (+) | | | (+) | | (+) |
| | | | LesFOM | | | + | + | (+) | (+) | | | | |
| 16 | 72 | M | NMB | Y | Y | + | | + | | | + | | |
| | | | LesPal | | | + | + | + | + | | | | |
| 17 | 84 | F | NFOM | – | – | + | | + | + | | | | |
| | | | LesFOM | | | + | + | + | + | | | | |
| 18 | 56 | F | NFOM | Y | Y | (+) | | + | | | + | | + |
| | | | LesFOM | | | + | + | + | + | | | (+) | (+) |
| 19 | 58 | M | NFOM | – | – | | | (+) | | | + | | |
| | | | LesFOM | | | | | | | | | | |
| 20 | 72 | M | NVT | Y | – | (+) | | + | | | + | | |
| | | | LesVT | | | | | (+) | (+) | | | | |
| 21 | 80 | M | NFOM | – | – | + | | + | (+) | | + | | |
| | | | LesFOM | | | (+) | (+) | (+) | (+) | | | | |
| 22 | 55 | M | NVT | Y | Y | | | + | | | + | | |
| | | | NBM | | | (+) | | + | | | + | | |
| | | | LesVT | | | + | + | + | + | | | | |
| 23 | 83 | F | NPal | Y | N | + | | + | | | + | | + |
| | | | LesPal | | | | | | | | | | |
| 24 | 74 | M | NFOM | – | – | | | + | + | | + | | |
| | | | LesFOM | | | | | | | | | | |
| 25 | 71 | M | NSPal | N | – | + | | | | | + | | + |
| | | | LesAlv | | | + | + | | | | + | | |
| 26 | 53 | F | NFOM | – | – | (+) | | + | (+) | | + | | + |
| | | | LesFOM | | | (+) | (+) | (+) | (+) | | | | |
| 27 | 61 | F | NVT | – | – | + | | + | | | + | | |
| | | | LesVT | | | (+) | (+) | + | + | | | (+) | (+) |
| 28 | 75 | F | NBM | N | N | | | | | | + | | |
| | | | LesBM | | | (+) | (+) | | | | | | |
| 29 | 62 | M | NFOM | – | – | | | | | | | | |
| | | | LesFOM | | | + | + | + | + | (+) | (+) | (+) | (+) |
| 30 | 75 | M | NVT | – | – | (+) | | + | (+) | | + | | |
| | | | LesFOM | | | + | + | + | + | | | | |
| | | | NBM | | | + | | + | | | + | | |
| 31 | 76 | M | NMB | N | N | | | | | | + | | |
| | | | LesPal | | | + | + | + | + | | | | |
| 32 | 73 | M | NFOM | (Y) | – | + | | + | | | | | |
| | | | LesFOM | | | + | + | | | | | | |

Age (years); Sex: M = Male, F = Female; Site: N = Normal; VT = Ventral tongue, Lat = Lateral tongue, FOM = Floor of mouth; BM = Buccal mucosa, Pal = Palate, Alv = Alveolus; Smoke/Alcohol: Y = Yes (Y) = formerly, N = No, – = Unknown; Keratin (K) identified in: B = Basal cells, S = Supbrabasal cells: + = most cells positive, (+) = few cells positive, blank = absent. CAM 5.2: see Methods.

**Table II** Assessment of keratin expression in normal oral mucosal biopsies from non-cancer patients

| | *Positive* | | *K8, K7* | | *K10* | | *K19* | | *K13* | |
|---|---|---|---|---|---|---|---|---|---|---|
| | *B* | *S* | *B* | *S* | *B* | *S* | *B* | *S* | *B* | *S* |
| NDT | + | + | (+) | – | – | – | + | (+) | – | + |
| NDT | + | + | (+) | – | – | – | + | – | – | + |
| NDT | + | + | (+) | – | – | (+) | + | – | – | + |
| NDT | + | + | (+) | – | – | (+) | + | – | – | + |
| NDT | + | + | (+) | – | – | (+) | + | (+) | – | + |
| NPal | + | + | (+) | – | – | (+) | (+) | – | – | (+) |
| NPal | + | + | (+) | – | – | + | (+) | – | – | (+) |
| NPal | + | + | (+) | – | – | + | – | – | – | – |
| NPal | + | + | (+) | – | – | (+) | (+) | – | – | (+) |
| NVT | + | + | – | – | – | – | + | – | – | + |
| NVT | + | + | – | – | – | – | + | – | – | + |
| NVT | + | + | – | – | – | – | + | (+) | – | + |
| NBM | + | + | – | – | – | (+) | – | – | – | + |
| NBM | + | + | (+) | – | – | (+) | + | – | – | + |
| NBM | + | + | (+) | – | – | – | (+) | – | – | + |
| NBM | + | + | (+) | – | – | – | + | – | – | + |
| NBM | + | + | + | – | – | – | + | – | – | + |
| NBM | + | + | – | – | – | – | + | – | – | + |
| NBM | + | + | (+) | – | – | (+) | + | – | – | + |
| NBM | + | + | (+) | – | – | – | + | – | – | + |

B = Basal; S = Suprabasal expression; Keratin present '+', absent '–', minimally expressed (+).

**Table III** Summary of keratin staining in clinically normal oral mucosal biopsies (–: absent; (+): few cells positive; +: most cells positive)

| | *Epithelial region* | *Staining pattern* | *K7/K8* | *K10* | *K19* | *K13* |
|---|---|---|---|---|---|---|
| *Non cancer patients* | Basal | – | 5 | 20 | 2 | 20 |
| | | (+) | 14 | 0 | 4 | 0 |
| | | + | 1 | 0 | 14 | 0 |
| | Suprabasal | – | 20 | 10 | 17 | 1 |
| | | (+) | 0 | 8 | 3 | 3 |
| | | + | 0 | 2 | 0 | 16 |
| *Oral cancer patients* | Basal | – | 13 | 33 | 8 | 33 |
| | | (+) | 9 | 1 | 5 | 1 |
| | | + | 12 | 0 | 21 | 0 |
| | Suprabasal | – | 33 | 26 | 22 | 3 |
| | | (+) | 0 | 3 | 6 | 2 |
| | | + | 1 | 5 | 6 | 29 |

current surgical practice now leads to a wider excision margin than practised previously. Thus the findings reported in the present study of inappropriate cytokeratin expression in 'normal' oral mucosa with no overt histomorphological signs of malignancy appear supportive of a field change.

### *Cytokeratin expression*

*Inappropriate expression of simple epithelial keratins* Keratins 8/7 (identified by CAM 5.2) were expressed in the basal cells of approximately a third of the normal biopsies from oral cancer patients. Keratins 8/K7 are not expressed by normal oral keratinocytes (Morgan *et al.*, 1987; Sawef *et al.*, 1991) although occasional staining of Merkel cells in the basal region has been observed (Morgan *et al.*, 1987). However the extent of K8/K7 expression in the basal cells reported above, together with the histomorphological detail was highly suggestive of basal cells expression of K8/K7 in normal oral mucosa of oral cancer patients. One study using *immunoblotting* techniques found K8 in basal cells of normal dorsal and ventral tongue (Clausen *et al.*, 1986) but this may have been due to 'contamination' by glandular tissue or even Merkel cells.

Previous reports have suggested that the simple epithelial keratins (such as K8) are only expressed in poorly differentiated tumours (Morgan *et al.*, 1987*a*). We have also found such expression in a significant number of well differentiated tumours (Ogden *et al.*, 1993). In so doing such basal cell expression mirrors that seen in the corresponding tumours (Morgan *et al.*, 1987*a*; Ogden *et al.*, 1993).

Further evidence supportive of a field change derives from the suprabasal expression of K19 in 'normal' buccal mucosa and floor of mouth region. Significantly such changes occurred in those sites most frequently affected by oral cancer (Mashberg & Samit, 1989). In most cases the corresponding tumours were also positive. It has been suggested that K19 expression, particularly in those oral sites where it is not usually seen, is related to inflammation (Bosch *et al.*, 1989). However we would challenge this since there was little evidence in most of our cases for profound inflammatory change. An increase in K19 expression within oral leukoplakias has been associated with mucosal instability and malignant change (Lindberg & Rheinwald, 1989). Since increased expression of K19 was not seen in the *non cancer* patients such a profile may herald a propensity tc undergo malignant change. However, loss of basal cell expression of

**Figure 2** Keratin 19 (LP2K) staining throughout the suprabasal epithelium of 'normal' floor of mouth (× 160). 1 cm bar = 62 μ.

**Figure 3** **a,** Loss of basal cell expression of K19 (LP2K) in 'normal' floor of mouth (× 140). 1 cm bar = 71 μ. **b,** Decreased basal cell expression of K19 (LP2K) in epithelium overlying tumour expressing K19 (× 140).

**Figure 4** Suprabasal expression of K10 (LH1) in a biopsy of 'normal' floor of mouth (× 140).

**Figure 5** Suprabasal expression of K10 (LH1) in 'normal' floor of mouth (× 140).

K19 in 'non-keratinising' sites was also identified, even in the mucosa above a K19 positive tumour (Figure 3c). Thus the significance of K19 expression (or lack of it) appears unclear.

*Reduction of appropriate cytokeratin expression* Further evidence for a field change comes from a reduction in complexity of differentiation. For example, as well as K19 reduction discussed above complete loss of K13 expression in 'normal' floor of mouth also occurred. A similar loss of K13 in 'normal' mucosa adjacent to a buccal mucosal cancer has been reported by Vaidya *et al.* (1989). Interestingly one patient with loss of K13 expression developed a recurrence one year later.

In Table I the tobacco and alcohol habits are recorded where known. Given that other important tumour diagnostic markers such as p53 can be influenced by smoking habits (Ogden *et al.*, 1992; Field *et al.*, 1992), the influence of tobacco on cytokeratin expression could be significant. For example, although there were approximately equal numbers of cases that were negative for CAM 5.2 in smokers compared to non-smokers, basal cell staining was much more frequent in the smoking group. There was no obvious association of tobacco or alchohol use with the other keratins studied. Furthermore, altered keratin profiles were also seen in those who did not smoke or take alcohol. Since further tumours can arise even in those abstaining from these high risk aetiological factors, the keratin profiles obtained offer a sensitive indication of altered tissue differentiation.

Whether these cases of inappropriate keratin expression are indicative of an increased likelihood of further tumours is not known, since this study only covers a 3 year period.

The changes in keratin expression reported above should not however be interepreted as inadequate excision of the primary lesion until their clinical significance is known. According to a recent review (Shaha *et al.*, 1988) multiple primary tumours occur in approximately 10% of all head and neck cancer patients, thus a clinically significant field change may not always occur.

Classical histopathological diagnosis relies upon the assessment of H & E stained sections to check that the margins are clear of tumour. The significance of these reported changes in keratin expression in essentially normal oral mucosa of oral cancer patients now requires evaluation. They may yet become a valuable additional test in the diagnostic and prognostic evaluation of patients with oral carcinomas.

This research is supported by the Cunningham Trust and Medical Research Council.

We thank Ms S. McQueen and Mr R. Kiddie for technical assistance and Mrs Dorothy Morrison for typing the manuscript.

### References

BOSCH, F.X., OUHAYOUN, J.P., BERNHARD, L., BADER, B.L., COLLIN, C., GRUND, C.I., LEE, I. & FRANKE, W.W. (1989). Extensive changes in cytokeratins expression patterns in pathologically affected human gingiva. *Virch. Archiv. B. Cell Pathol.*, **58**, 59–77.

CLAUSEN, H., MOE, D., BUSCHARD, K. & DABELSTEEN, E. (1986). Keratin proteins in human oral mucosa. *J. Oral Pathol.*, **15**, 36–42.

COOPER, D., SCHERMER, A., SUN, T.-T. (1985). Classification of human epithelia and their neoplasms using monoclonal antibodies to keratins: strategies, applications and limitations. *Lab. Invest.*, **52**, 243–256.

COWPE, J.G., LONGMORE, R.B. & GREEN, M.W. (1988). Quantitative exfoliative cytology of abnormal oral mucosal smears. *J. Roy. Soc. Med.*, **81**, 509–513.

FIELD, J.K., SPANDIDOS, D.A. & STELL, P.M. (1992). Overexpression of p53 gene in head and neck cancer linked with heavy smoking and drinking. *Lancet*, **339**, (8791), 502–503.

INCZE, J., VAUGHAN, C.W., LIU, P., STRONG, M.S. & KULAPADITHAR, O.M.B. (1982). Premalignant changes in normal appearing epithelium in patients with squamous cell carcinoma of the upper aerodigestive tract. *Am. J. Surg.*, **144**, 401–405.

LANE, E.B. & ALEXANDER, C.M. (1990). Use of keratin antibodies in tumour diagnosis. *Sem. Cancer Biol.*, **1**, 165–179.

LINDBERG, K. & RHEINWALD, J.G. (1989). Suprabasal 40 kd keratin (K19) expression as an immunohistologic marker of premalignancy in oral epithelium. *Am. J. Pathol.*, **134**, 89–98.

LIPPMAN, S.M. & HONG, W.K. (1989). Second malignant tumours in head and neck squamous cell carcinoma: the overshadowing threat of patients with early-stage disease. *Int. J. Rad. Oncol. Biol. Phys.*, **17**, 691–694.

MAKIN, C.A., BOBROW, L.G. & BODMER, W.F. (1984). Monoclonal antibody to cytokeratin for use in routine histopathology. *J. Clin. Pathol.*, **37**, 975–983.

MASHBERG, A. & SAMIT, A.M. (1989). Early detection, diagnosis and management of oral and oropharyngeal cancer. *CA – a cancer journal for clinicians*, **39**, 67–88.

MORGAN, P.R., LEIGH, I.M., PURKIS, P.E., GARDNER, I.D., VAN MUIJEN, G.N.P. & LANE, E.B. (1987). Site variation in keratin expression in human oral epithelia – an immunocytochemical study of individual keratins. *Epithelia*, **1**, 31–43.

MORGAN, P.R., SHIRLAW, P.J., JOHNSON, N.W., LEIGH, I.M. & LANE, E.B. (1987*a*). Potential applications of antikeratin antibodies in oral diagnosis. *J. Oral Pathol.*, **16**, 212–222.

OGDEN, G.R., COWPE, J.G. & GREEN, M.W. (1990). The effect of distant malignancy upon quantitative cytologic assessment of normal oral mucosa. *Cancer*, **65**, 477–480.

OGDEN, G.R., COWPE, J.G. & GREEN, M.W. (1990). Evidence of field change in oral cancer. *Br. J. Oral Maxillofac. Surg.*, **28**, 390–392.

OGDEN, G.R. (1991). Second malignant tumours in head and neck cancer. *Br. Med. J.*, **302**, 193–194.

OGDEN, G.R., COWPE, J.G. & GREEN, M.W. (1991). Detection of field change in oral cancer using oral exfoliative cytologic study. *Cancer*, **68**, 1611–1615.

OGDEN, G.R., NAIRN, A., CARMICHAEL, A., COGHILL, G., CREE, I.A., GREEN, M.W., HOPWOOD, D.V. & CHISHOLM, D.M. (1992). Preservation of keratin expression in oral mucosa using a novel transport medium. *J. Oral. Pathol. Med.*, **21**, 17–20.

OGDEN, G.R., KIDDIE, R.A., LUNNY, D.P. & LANE, D.P. (1992). Assessment of p53 protein expression in normal, benign, and malignant oral mucosa. *J. Pathol.*, **166**, 389–394.

OGDEN, G.R., CHISHOLM, D.M., ADI, M. & LANE, E.B. (xxxx). Cytokeratin expression in oral cancer and its relationship to tumour differentiation. *J. Oral Pathol Med.*, (in press).

SAWAF, M.H., OUHAYOUN, J.P. & FOREST, N. (1991). Cytokeratin profiles in oral epithelia: A review and a new classification. *J. Biol. Buccale*, **19**, 187–198.

SHAHA, A., HOOVER, E., MARTI, J. & KRESPI, Y. (1988). Is routine triple endoscopy cost-effective in head and neck cancer? *Am. J. Surg.*, **155**, 750–753.

SLAUGHTER, D.P. (1946). Multicentric origin in intra oral carcinoma. *Surgery*, **20**, 133–146.

SLAUGHTER, D.P., SOUTHWICK, H.W. & SMEJKAL, W. (1953). Field cancerization in oral stratified squamous epithelium. *Cancer*, **6**, 963–968.

SMEDTS, F., RAMAEKERS, F.C.S., ROBBEN, H., PRUSZCYNSKI, M., VAN MUIJEN, G., LANE, B., LEIGH, I. & VOOIJS, P. (1990). Changing patterns of keratin expression during progression of cervical intraepithelial neoplasia. *Am. J. Pathol.*, **136**, 657–668.

STRONG, M.S., INCZE, J. & VAUGHAN, C.W. (1984). Field cancerization in the aerodigestive tract – its etiology, manifestation and significance. *J. Otolaryngol.*, **13**, 1–6.

VAIDYA, M.M., BORGES, A.M., PRADHAM, S.A., RAJPAL, R.M. & BHISSEY, A.N. (1989). Altered keratin expression in buccal mucosal squamous cell carcinoma. *J. Oral Pathol. Med.*, **18**, 282–286.

WRIGHT, A. & SHEAR, M. (1985). Epithelial dysplasia immediately adjacent to oral squamous cell carcinomas. *J. Oral Pathol.*, **14**, 559–564.

WYNDER, E.G., DODO, H., BLOCH, D., GRANT, R.C. & MOORE, O.S. (1969). Epidemiologic investigation of multiple primary cancer of the upper alimentary and respiratory tracts. *Cancer*, **24**, 730–739.